# Anti-inflammatory effect of sodium butyrate preconditioning during myocardial ischemia/reperfusion

**DOI:** 10.3892/etm.2014.1726

**Published:** 2014-05-20

**Authors:** XIAORONG HU, KAI ZHANG, CHANGWU XU, ZHIQAING CHEN, HONG JIANG

**Affiliations:** 1 Department of Cardiology, Renmin Hospital of Wuhan University, Cardiovascular Research Institute of Wuhan University, Wuhan, Hubei 430060, P.R. China; 2 Department of Cardiology, Huangshi Central Hospital, Affiliated Hospital of Hubei Polytechnic University, Huangshi, Hubei 435000, P.R. China

**Keywords:** sodium butyrate, high mobility group box 1 protein, myocardial ischemia, reperfusion

## Abstract

High mobility group box 1 protein (HMGB1) has an important role in myocardial ischemia/reperfusion (I/R) injury. Sodium butyrate, an inhibitor of histone deacetylase, has been shown to inhibit HMGB1 expression. In the present study, the effect of sodium butyrate on myocardial I/R injury in rats was investigated. Anesthetized male rats were intraperitoneally administered sodium butyrate (100 or 300 mg/kg) 30 min prior to the induction of ischemia. The rats were then subjected to ischemia for 30 min followed by reperfusion for 4 h. Infarct size, lactate dehydrogenase (LDH), creatine kinase (CK) and superoxide dismutase (SOD) activity and malondialdehyde (MDA) levels were then measured. The expression of HMGB1 was assessed using western blot analysis. The results demonstrated that pretreatment with sodium butyrate (300 mg/kg) significantly reduced the infarct size, as well as the levels of LDH and CK (P<0.05). In addition, sodium butyrate (300 mg/kg) was shown to significantly inhibit the I/R-induced increase in the level of MDA and reduction in the level of SOD (P<0.05). Furthermore, treatment with sodium butyrate (300 mg/kg) was found to significantly inhibit the expression of TNF-α, IL-6 and HMGB1 induced by I/R injury (P<0.05). In conclusion, the results from the present study suggest that preconditioning with sodium butyrate may attenuate myocardial I/R injury by inhibition of the expression of inflammatory mediators during myocardial I/R.

## Introduction

Myocardial ischemia/reperfusion (I/R) injury is common following acute coronary syndrome and heart transplantation. Although reperfusion is essential for the survival of ischemic myocardial tissue, reperfusion causes additional cellular injury. I/R may result in local myocardial inflammation, accompanied by apoptosis, which may cause cardiomyocyte damage (1,2). Cardiomyocyte damage is thought to occur as a result of an intense inflammatory response initiated by the infiltration of leukocytes and the production of pro-inflammatory cytokines. High mobility group box 1 protein (HMGB1), a non-chromosomal nuclear protein, has been identified as a novel pro-inflammatory cytokine that functions as a late mediator of inflammation in sepsis, acute lung injury, autoimmune disease and coronary artery diseases (3–6). Previous studies have shown that HMGB1 acts as an early mediator of inflammation and promotes cell injury during myocardial I/R. HMGB1 also promotes the release of early pro-inflammatory cytokines, including tumor necrosis factor-α (TNF-α) and interleukin-6 (IL-6). Inhibition of HMGB1 by HMGB1 A box peptide (a specific HMGB1 antagonist) has been shown to have a protective effect against myocardial I/R injury and to inhibit the release of TNF-α and IL-6 (7,8). These results suggest that HMGB1 may have an important role in myocardial I/R injury.

Sodium butyrate, an inhibitor of histone deacetylase, has been previously shown to have an anti-inflammatory effect and to inhibit the expression of HMGB1 in an ischemic model of stroke (9,10). Therefore, in the present study, it was hypothesized that sodium butyrate may protect against myocardial I/R injury by inhibiting the expression of HMGB1. In the present study the effect of sodium butyrate preconditioning on myocardial I/R injury in a rat myocardial I/R model was investigated.

## Materials and methods

### Animal preparation and experimental design

The experimental protocol was in accordance with the Guidelines for the Care and Use of Laboratory Animals published by the US National Institutes of Health (Bethesda, MD, USA) and was approved by the Institutional Animal Care and Use Committee (Renmin Hospital of Wuhan University, Wuhan, China). Male Sprague-Dawley rats (250–300 g) were randomly divided into four groups receiving the following treatments: group 1, sham-operated control (SO; n=10): rats were subjected to surgical manipulation without the induction of myocardial ischemia; group 2, I/R group (n=15): rats were subjected to left anterior descending coronary artery (LAD) occlusion for 30 min followed by reperfusion for 4 h; group 3, SB1 + I/R (SB1-I/R; n=15): rats were administered sodium butyrate (100 mg/kg) dissolved in sterile saline intraperitoneally 30 min prior to LAD occlusion; and group 4, SB2 + I/R (SB2-I/R; n=15): rats were administered sodium butyrate (300 mg/kg) intraperitoneally 30 min prior to LAD occlusion.

Following anesthetization with sodium pentobarbital (45 mg/kg, intraperitoneally), the rats were ventilated artificially using a volume-controlled rodent respirator at 70 strokes/min. The rats were placed on an electric heating pad to maintain their body temperature at 37°C. Heparin (200 IU/kg) was then administered intravenously prior to the induction of ischemia. Lead-II of an electrocardiogram was monitored with subcutaneous stainless steel electrodes. The electrocardiogram was monitored using a computer-based EP system (LEAD2000B; Jinjiang Ltd., Chengdu, China).

A thoracotomy through a left parasternal incision was performed. The pericardium was incised, and the anterior wall of the left ventricle was exposed. A 4-0 silk suture on a small curved needle was passed through the myocardium beneath the middle segment of the LAD branch coursing down the middle of the anterior wall of the left ventricle. A small vinyl flake was passed into the ends of the suture, which was then fixed by clamping the tube with a mosquito hemostat. A successful myocardial I/R model was confirmed by changes in the ST segment elevation in Leads-II and regional cyanosis of the myocardial surface. The rats then underwent a 30-min LAD occlusion, followed by a 4-h reperfusion.

### Assessment of myocardial injury

To assess the lactate dehydrogenase (LDH) and creatine kinase (CK) activities, blood samples were collected, centrifuged and stored at −20°C until analysis. The samples were analyzed using standard techniques using an LDH Assay kit and CK Assay kit in accordance with the manufacturer’s instructions (Nanjing Jiancheng Bioengineering Institute, Nanjing, China). Values were expressed as international units (IU) per liter.

### Assessment of infarct size

Following the 4-h reperfusion, the LAD was again occluded and 1.5% Evans blue dye (2 ml) was injected via the femoral vein. The risk area was analyzed using negative staining with Evans blue. The rats were then sacrificed and their hearts were excised and frozen overnight. The atria and right ventricle were removed and the left ventricle was sectioned into 2-mm thick transverse slices from the apex to base. The risk area was separated from the colored nonischemic area (blue) and then incubated with a 1% solution of 2,3,5-triphenyltetrazolium chloride (TTC, in 0.2 M Tris buffer, pH 7.4) stain for 20 min at 37°C. Viable myocardium was stained red by TTC, whilst necrotic myocardium was not stained red. In each slice, the infarct size and the risk area (left ventricular areas) were determined using a computer-assisted image analysis system (Image-Pro Plus 3.0, Media Cybernetics Inc., Rockville, MD, USA) and multiplied by the thickness of the slice to calculate the volume of the risk area. The infarct size was expressed as a percentage of the risk area volume (infarct size/risk area).

### Analysis of myocardial TNF-α and IL-6 expression

The expression levels of TNF-α and IL-6 in myocardial tissue supernatants were determined using a commercial enzyme-linked immunosorbent assay kit (Nanjing Jiancheng Bioengineering Institute, Nanjing, China) in accordance with the manufacturer’s instructions. The sensitivity of the assay was 1 pg/ml for TNF-α and IL-6.

### Measurement of myocardial malondialdehyde (MDA) levels and superoxide dismutase (SOD) activity

The concentration of MDA and the activity of SOD in myocardial tissue were measured using an MDA Assay kit and SOD Assay kit in accordance with the manufacturer’s instructions (Nanjing Jiancheng Bioengineering Institute) as previously described (11). The MDA concentration and SOD activity were used to indicate the amount of oxygen free radicals and the lipid superoxide level in the myocardium, respectively.

### Western blot analysis

The protein expression of HMGB1 in the pulverized frozen ischemic area of the left ventricle or cultured myocardium was analyzed using quantitative immunoblotting using an antibody against HMGB1 (Santa Cruz, Santa Cruz Biotechnology, Inc., CA, USA) as previously described (11). The expression level was normalized against glyceraldehyde-3-phosphate dehydrogenase (GAPDH) expression.

### Statistical analysis

All values are presented as the mean ± standard deviation. The student t-test was used for comparisons between the groups. A one-way analysis of variance (ANOVA) or Welch ANOVA was used for comparisons among the groups and the Student-Newman-Keuls or Dunnett T3 test was used for post-hoc multiple comparisons. P<0.05 was considered to indicate a statistically significant difference.

## Results

### Infarct size

**Figure 1:**
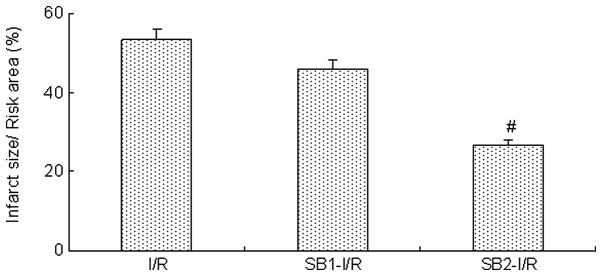
Effect of sodium butyrate on the infarct size during I/R (n=5 for each group). ^#^P<0.05, vs. the I/R group. I/R, ischemia/reperfusion; SB1, sodium butyrate (100 mg/kg); SB2, sodium butyrate (300 mg/kg).

Following the 4-h reperfusion, treatment with sodium butyrate (300 mg/kg) was found to reduce the infarct size induced by myocardial I/R compared with that in the I/R group (26.8±3.8 vs. 53.4±4.9%; P<0.05). However, a lower dose of sodium butyrate (100 mg/kg) did not have an inhibitory effect (P>0.05; Fig. 1)

### LDH and CK activities

**Figure 2:**
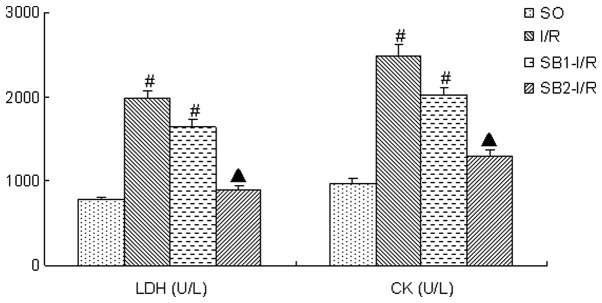
Effect of sodium butyrate on LDH and CK during I/R. (n=10 or 15 for each group). ^#^P<0.05 vs. the SO group; ^▲^P<0.05 vs. I/R group. LDH, lactate dehydrogenase; CK, creatine kinase; SO, sham-operated control; I/R, ischemia/reperfusion; SB1, sodium butyrate (100 mg/kg); SB2, sodium butyrate (300 mg/kg).

Following the 4-h reperfusion, the LDH and CK activities in the I/R group were significantly increased compared with those in the SO group (P<0.05). Treatment with a high dose of sodium butyrate (300 mg/kg) significantly inhibited the increase of LDH and CK levels (P<0.05); however, treatment with 100 mg/kg sodium butyrate did not provide an inhibitory effect (P>0.05; Fig. 2)

### TNF-α and IL-6 levels

**Figure 3:**
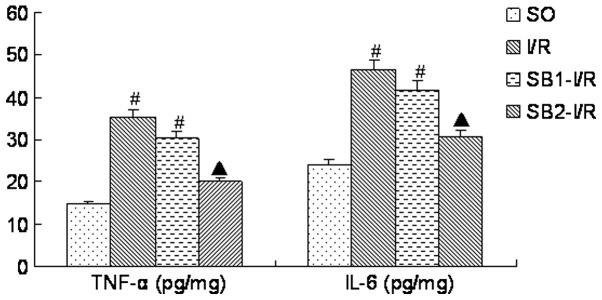
Effect of sodium butyrate preconditioning on TNF-α and IL-6 during I/R (n=5 for each group). ^#^P<0.05 vs. the SO group; ^▲^P<0.05 vs. the I/R group. TNF-α, tumor necrosis factor-α; IL-6, interleukin-6; SO, sham-operated control; I/R, ischemia/reperfusion; SB1, sodium butyrate (100 mg/kg); SB2, sodium butyrate (300 mg/kg).

Following the 4-h reperfusion, the levels of TNF-α and IL-6 in the I/R group were significantly increased compared with those in the SO group (P<0.05). Treatment with a high dose of sodium butyrate (300 mg/kg) significantly inhibited the increases of the TNF-α and IL-6 levels (P<0.05), whilst treatment with 100 mg/kg sodium butyrate did not provide a protective effect (P>0.05; Fig. 3).

### MDA and SOD levels

**Figure 4:**
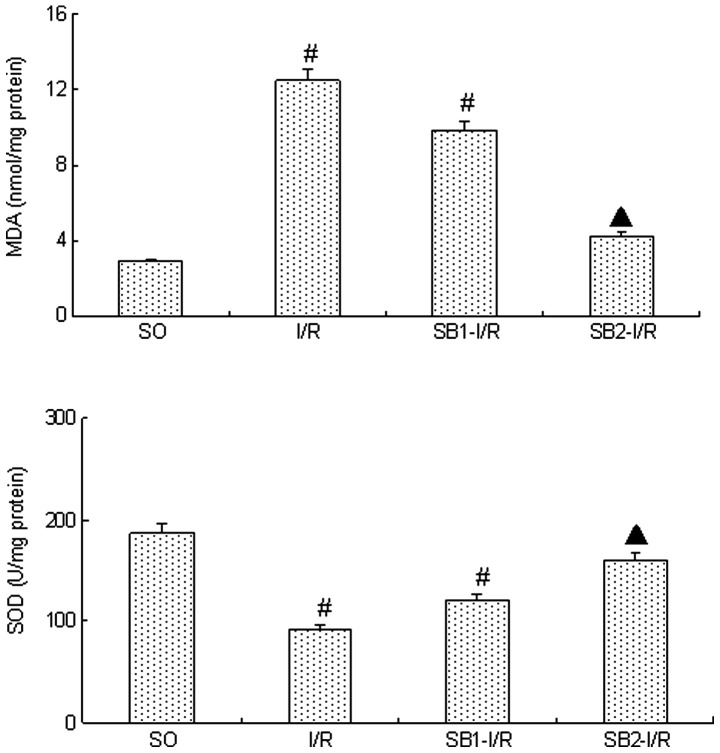
Effect of sodium butyrate on MDA and SOD during I/R (n=5 for each group). ^#^P<0.05 vs. the SO group; ^▲^P<0.05 vs. the I/R group. MDA, malondialdehyde; SOD, superoxide dismutase; SO, sham-operated control; I/R, ischemia/reperfusion; SB1, sodium butyrate (100 mg/kg); SB2, sodium butyrate (300 mg/kg).

Following the 4-h reperfusion, the level of MDA in the I/R group was significantly increased whilst the level of SOD decreased significantly compared with those in the SO group (P<0.05). Sodium butyrate (300 mg/kg) was found to significantly inhibit the increase in the MDA levels and the reduction in the SOD levels (P<0.05). However, treatment with a lower dose of sodium butyrate (100 mg/kg) did not provide a protective effect (P>0.05; Fig. 4).

### Effect of sodium butyrate on HMGB1 expression

**Figure 5:**
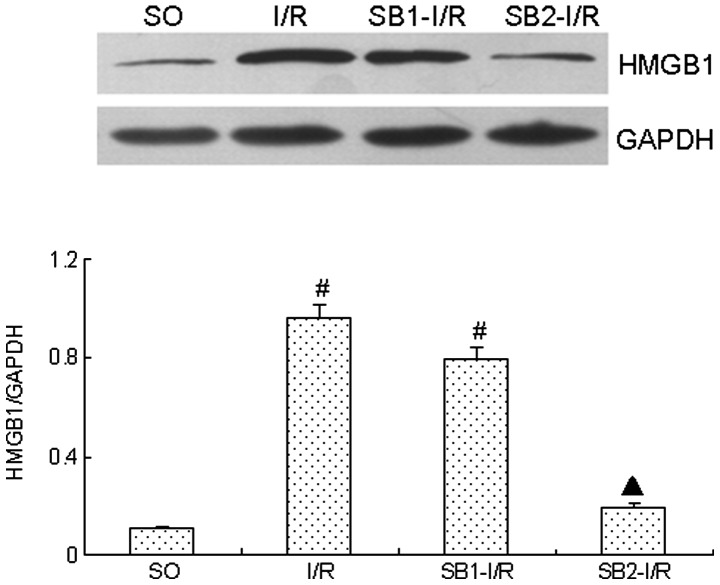
Effect of sodium butyrate on HMGB1 expression during I/R (n=5 for each group). ^#^P<0.05 vs. the SO group; ^▲^P<0.05 vs. the I/R group. HMGB1, high mobility group box 1 protein; SO, sham-operated control; I/R, ischemia/reperfusion; SB1, sodium butyrate (100 mg/kg); SB2, sodium butyrate (300 mg/kg).

Following the 4-h reperfusion, HMGB1 expression was markedly increased compared with that in SO group (P<0.05). However, treatment with sodium butyrate (300 mg/kg) significantly inhibited the expression of HMGB1 (P<0.05). By contrast, treatment with a lower dose of sodium butyrate (100 mg/kg) did not provide an inhibitory effect (P>0.05; Fig. 5).

## Discussion

HMGB1 has been shown to function as a novel pro-inflammatory cytokine with an important role in myocardial I/R injury (7,8). Previous studies have shown that there is cross-talk between HMGB1 and other pro-inflammatory cytokines, including TNF-α, IL-6 and C-reactive protein (CRP) (7,12–15). HMGB1, when it is released from necrotic cells, apoptotic cells, macrophages or monocytes, upregulates the levels of IL-1, IL-6, TNF-α, CRP and macrophage inflammatory proteins (MIP-1α and MIP-1β). Other pro-inflammatory cytokines may in turn promote the release of HMGB1 (7,12–15), indicating that this mechanism reinforces the inflammatory process. In addition, our previous study demonstrated that HMGB1 may promote the apoptosis of myocardium in a dose-dependent manner (11). Inflammation and apoptosis have a critical role in myocardial I/R injury (1,2). In the present study, pretreatment with sodium butyrate (300 mg/kg) was found to significantly attenuate myocardial I/R injury, as well as downregulate the expression of TNF-α, IL-6 and HMGB1. Previous studies have shown that sodium butyrate is also able to inhibit HMGB1 expression in other diseases (9,10). Therefore, in the present study, it was hypothesized that sodium butyrate may protect against myocardial I/R injury and inflammation by inhibiting HMGB1 expression.

In addition, the present study demonstrated that pretreatment with sodium butyrate (300 mg/kg) decreases the levels of MDA (a reactive oxygen species) and increases the levels of SOD (a key antioxidant enzyme). Previous studies have shown that reactive oxygen species may be involved in the release of the pro-inflammatory cytokine HMGB1. Tang *et al* (16) demonstrated that hydrogen peroxide, a reactive oxygen species, stimulates the release of HMGB1 from macrophages and monocytes. Furthermore, Tsung *et al* (17) showed that HMGB1 released from cultured hepatocytes was an active process regulated by reactive oxygen species. Zhang *et al* (18) showed that antioxidants inhibit HMGB1 expression and reduce pancreatic injury in rats with severe acute pancreatitis, and this was mainly attributed to the release of HMGB1 (19,20), further indicating that inhibiting reactive oxygen species may inhibit HMGB1 expression. These results suggest that pretreatment with sodium butyrate inhibits HMGB1 expression, which may be associated with the inhibition of reactive oxygen species induced by myocardial I/R injury.

In conclusion, the results from the present study suggest that preconditioning with sodium butyrate (300 mg/kg) is able to attenuate myocardial I/R injury, which may be associated with the inhibition of the expression of inflammatory mediators during myocardial I/R.
